# Glucagon-Like Peptide-1 Triggers Protective Pathways in Pancreatic Beta-Cells Exposed to Glycated Serum

**DOI:** 10.1155/2013/317120

**Published:** 2013-04-29

**Authors:** Alessandra Puddu, Roberta Sanguineti, Arianna Durante, Alessio Nencioni, François Mach, Fabrizio Montecucco, Giorgio L. Viviani

**Affiliations:** ^1^Department of Internal Medicine and Medical Specialties, University of Genova, 6 Viale Benedetto XV, 16132 Genova, Italy; ^2^Division of Cardiology, Geneva University Hospitals, Faculty of Medicine, Foundation for Medical Researches, 64 Avenue de la Roseraie, 1211 Geneva, Switzerland

## Abstract

Advanced glycation end products (AGEs) might play a pathophysiological role in the development of diabetes and its complications. AGEs negatively affect pancreatic beta-cell function and the expression of transcriptional factors regulating insulin gene. Glucagon-like peptide-1 (GLP-1), an incretin hormone that regulates glucose homeostasis, might counteract the harmful effects of AGEs on the beta cells in culture. The aim of this study was to identify the intracellular mechanisms underlying GLP-1-mediated protection from AGE-induced detrimental activities in pancreatic beta cells. HIT-T15 cells were cultured for 5 days with glycated serum (GS, consisting in a pool of AGEs), in the presence or absence of 10 nmol/L GLP-1. After evaluation of oxidative stress, we determined the expression and subcellular localization of proteins involved in maintaining redox balance and insulin gene expression, such as nuclear factor erythroid-derived 2 (Nrf2), glutathione reductase, PDX-1, and MafA. Then, we investigated proinsulin production. The results showed that GS increased oxidative stress, reduced protein expression of all investigated factors through proteasome activation, and decreased proinsulin content. Furthermore, GS reduced ability of PDX-1 and MafA to bind DNA. Coincubation with GLP-1 reversed these GS-mediated detrimental effects. In conclusion, GLP-1, protecting cells against oxidants, triggers protective intercellular pathways in HIT-T15 cells exposed to GS.

## 1. Introduction

Pancreatic beta cell dysfunction is a key pathophysiological target in diabetes mellitus [1–3]. The concept that glucose via glycation as well as glucotoxicity is one of the main damaging molecules is widely accepted [4, 5]. Furthermore, hyperglycemia increases the production of AGEs, a group of compounds derived from the nonenzymatic reaction between reducing sugars and proteins, lipids, and DNA [6]. It is well known that a long-lasting deleterious effect of hyperglycemia persists independently of the level of glucose [7–9]. This “memory” might be explained by the persistent overproduction of reactive oxygen species (ROS) directly induced by AGEs via the activation of their receptors [10]. Furthermore, the increase in pancreatic beta-cell responsiveness to oxidants [11, 12] might result in a decreased nuclear availability of the regulators of insulin promoters PDX-1 (pancreatic and duodenal homeobox-1) and MafA (v-maf musculoaponeurotic fibrosarcoma oncogene homologue A) [13–16]. Recently, we also showed that exposure of pancreatic beta-cells to AGEs decreased glutathione (GSH) availability and negatively affected expression and subcellular localization of PDX-1 [11, 16]. Since GSH is a pivotal antioxidant factor [17] regulated via the new synthesis of GSH from GSSG (glutathione disulphide) by glutathione reductase (GSR), we also focused on these molecular mechanisms. It has been reported that both GSH synthesis and GSR expression are regulated by nuclear factor erythroid 2 p45-related factor 2 (Nrf2), a basic leucine zipper transcription factor that in response to oxidative stress translocates to the nucleus and binds to antioxidant-response elements (AREs) in the promoters of target genes [18, 19]. Interestingly, it has been also reported that Nrf2 is upregulated by analogues of glucagon-like peptide-1 (GLP-1) [20]. Given the potential regulatory activity of GLP-1 (an incretin hormone that participates to glucose homeostasis [21]), the aim of the present study was to identify the potential protective pathways triggered by GLP-1 to counteract pancreatic beta-cell dysfunction mediated by glycated serum (GS).

## 2. Materials and Methods

### 2.1. Cell Culture and Stimulation

The hamster pancreatic beta-cell line, HIT-T15, was purchased from the American Type Culture Collection (Manassas, VA, USA). These cells were grown in RPMI 1640 medium supplemented with 10% FBS, 4 mM L-glutamine, 100 IU penicillin-G, and 100 *μ*g/mL streptomycin at 37°C in a humidified atmosphere of 5%  CO_2_. Culture media were replaced every 2 days. When cells reached confluence, they were passaged after trypsin-EDTA detachment and seeded in multiwell plates for various experiments. Then, HIT-T15 cells were plated in 6-well dishes (7 × 10^5^ cells per well) and incubated for 5 days in media containing GS in the presence or absence of 10 nmol/L GLP-1. The investigation protocol was conformed to the principles outlined in the Declaration of Helsinki.

### 2.2. Preparation of GS

GS was prepared by incubation at 37°C for 7 days of heat-inactivated FBS with 50 mmol/L ribose. The serum was then extensively dialyzed against 0.1 M PBS, pH 7.4, to remove surplus sugar. Aliquots of FBS were processed in the same way but without ribose (NGS) and were used for standard culture (CTR). Content of pentosidine, a well-known marker of glycoxidative stress, was evaluated by HPLC and used as a measure of proteins' glycation [22]. The concentration of pentosidine in the experimental medium containing GS ranged between 1.5 and 4 × 10^5^ pmol/l, which corresponds to the plasma levels of pentosidine detected in the plasma of diabetic patients [23].

### 2.3. Reactive Oxygen Species Detection

Intracellular reactive oxygen species (ROS) level was measured using the cell-permeable fluorescent probe, 2′,7′-dichlorofluorescein diacetate (DCFH-DA) (Sigma-Aldrich, Milan, Italy). In brief, cells were seeded into 6-well culture plates at 7 × 10^5^ cells/well and treated for 5 days with GS with or without GLP-1 then washed twice with Hank's Buffered Salt Solution (HBSS) and incubated with fresh DCFH-DA (10 *μ*M) in HBSS for 30 min at 37°C in 5% CO_2_. DCFH-DA stock solution (20.5 mM) was prepared in DMSO and stored at −20°C for maximum one month. After that, cells were washed twice in HBSS, and wells were filled with 1 mL HBSS before fluorescence acquisition in a plate reader (TECAN InfinitePro200) (Ex: *λ*485/Em: *λ*535 nm). Fluorescent emission was normalized to total protein content.

### 2.4. Reverse Transcriptase Polymerase Reaction

Total RNA was extracted from HIT-T15 with RNeasy kit (QIAGEN s.r.l., Milan, Italy) according to manufacturer's instructions. The RNA concentrations were determined spectrophotometrically and equal quantities of total RNA were used from different samples. One microgram of RNA was reverse transcripted to cDNA using GoScript Reverse Transcription System (PROMEGA ITALIA, Milan, Italy) and then amplified by PCR. As reported in Table 1 all the samples were amplified in a linear amplification range established using a serial cDNA dilution and varying the number of cycles (28 cycles for Actin, Preproinsulin and PDX-1; 40 cycles for Nrf2). PCR products were electrophoresed onto a 1.5% agarose gel containing EuroSafe Nucleic Acid Stain (EUROCLONE S.p.A, Milan, Italy) and visualized under UV light. The relative intensities of the bands were quantified by densitometric analysis. 

### 2.5. Cell Lysis and Subcellular Fractionation

At the end of the experiments, a set of HIT-T15 cells were lysed in RIPA buffer (50 mmol/L Tris HCl pH 7.5, 150 mmol/L NaCl, 1% NP40, 0.1% SDS), supplemented with protease and phosphatase inhibitors. Another set of HIT-T15 cells was processed for subcellular fractionation using the Subcellular Protein Fractionation Kit (Pierce Biotechnology, Rockford, IL, USA) according to the manufacturer's instructions. Briefly, various cellular compartments were isolated by sequential addition of different extraction buffers to the cell pellet. Each subcellular fraction was collected after centrifugation and stored at −80°C. Nuclear soluble and chromatin-bound protein extracts obtained from each experimental condition were used for immunoblot analysis. Protein concentration of each sample was determined using BCA Protein Assay Kit (Pierce Biotechnology, Rockford, IL, USA).

### 2.6. Immunoblot

Thirty micrograms of total cell lysate were separated on an SDS-PAGE and transferred onto nitrocellulose. Filters were blocked in 5% BSA and incubated overnight at 4°C with primary specific antibodies (against: GSR, Nrf2, and *β*-Actin from Santa Cruz Biotechnology, Inc. Santa Cruz, CA, USA; against: PDX-1 from Millipore, Billerica, MA, USA; against MafA from Bethyl Lab.). Secondary specific horseradish-peroxidase-linked antibodies were added for 1 h at room temperature. Bound antibodies were detected using an enhanced chemiluminescence lighting system (Luminata Classico, Millipore, Billerica, MA, USA), according to manufacturer's instructions. Bands of interest were quantified by densitometry using the NIH program ImageJ. To verify equal loading of the proteins, membranes were stripped again, reblocked, and reprobed to detect *β*-actin. Values of proteins of interest were normalized to total amounts of *β*-actin and expressed as percentages of NGS control (defined as 100%).

### 2.7. siRNA

For the GLP-1R siRNA experiment a pool of 4 prevalidated siRNAsdesigned for hamster GLP-1R were used (Riboxx GmbH, Radebeul, Germany). HIT-T15 cells were seeded in 12-well plates in culture medium without antibiotics and grown overnight to reach 40% confluency. The next day, FECT-siRNA complexes were prepared according to manufacturer's instructions. Cells were transfected with 20 nmol/L GLP-1RsiRNA or control siRNA (which correspond to a nontargetting 23-nucleotide siRNA designed as negative control) for 24 h before switching to fresh culture medium. Forty-eight hours after transfection cells were incubated for 5 days in media containing GS and GLP-1. We tested GLP-1R protein level by immunoblotting. Transfection results in 50% knockdown of GLP-1R at 48 h, and 7 days after transfection.

### 2.8. Proinsulin Content

To evaluate proinsulin content another set of cells, grown in the same conditions, was washed twice with PBS, pH 7.4, at 0°C, extracted with acid/ethanol (0.15 mol/L HCl in 75% ethanol in H_2_O) for 16 h at 0°C, then centrifuged at 15,000 ×g at 4°C. Supernatants were collected and stored at –20°C until the proinsulin determination was performed by ELISA (Mercodia AB, Uppsala, Sweden). The results were normalized to total protein concentration.

### 2.9. Statistical Analysis

The results are representative of at least 3 experiments. All analyses were carried out with the GraphPad Prism 4.0 software (GraphPad Software, San Diego, CA, USA). Data were expressed as the mean±SE and then analysed using Student's *t*-test. *P* value <0.05 was considered as statistically significant.

## 3. Results

### 3.1. GLP-1 Reduces GS-Mediated ROS Release

**Figure 1:**
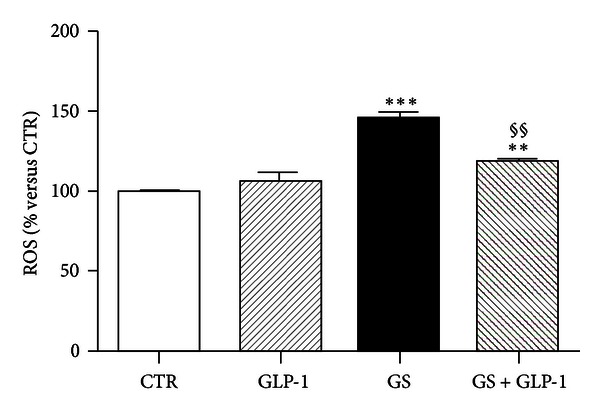
GLP-1 abrogates AGE-induced intracellular ROS production. After treatment for 5 days in standard medium (CTR) or in medium containing AGEs (GS) in the presence or absence of 10 nmol/L GLP-1, HIT-T15 cells were prelabeled with DCFH-DA for 30 min and fluorescence was analyzed. Data were expressed as the mean ± SE of four independent experiments. **P < 0.01 and ***P < 0.001 versus CTR; ^§§^ *P* < 0.01 versus GS.

Exposure of HIT-T15 cells to GS significantly increased (by 1.5-fold) the release of ROS as compared to control (CTR). Coincubation with GLP-1 abrogated GS-mediated ROS production (Figure 1).

### 3.2. GLP-1 Restores Nrf2 Protein Levels in Pancreatic Beta-Cells Exposed to GS

**Figure 2:**
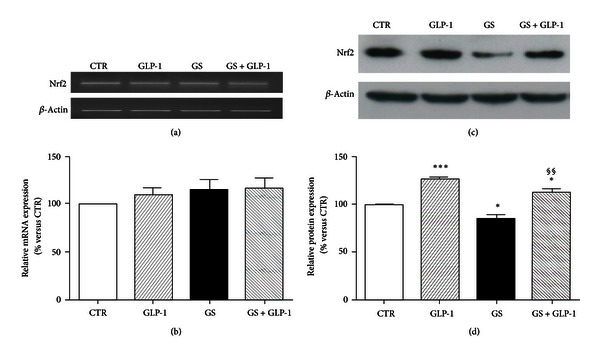
GLP-1 restores Nrf2 protein levels in HIT-T15 cells cultured in the presence of GS. (a)-(b) Semiquantitative RT-PCR of Nrf2 mRNA expression in HIT-T15 cells cultured 5 days in standard medium (CTR) or in medium containing AGEs (GS) in the presence or absence of 10 nmol/L GLP-1. (a) Representative agarose gel of three different experiments. (b) Quantification of densitometries of agarose gel bands. Data were represented as mean±SE of percentage change from CTR level (100%) (*n* = 3). (c)-(d) Western blot analysis of Nrf2 protein intracellular expression in HIT-T15 cells cultured in the same conditions as mRNA experiments. (c) Representative western blot of three different experiments. (d) Quantification of densitometries of western blot bands. Data were expressed as mean ± SE of fold induction relative to *β*-actin (*n* = 3). *P < 0.05 versus CTR; ***P < 0.001 versus CTR; ^§§^ *P* < 0.01 versus GS.

**Figure 3:**
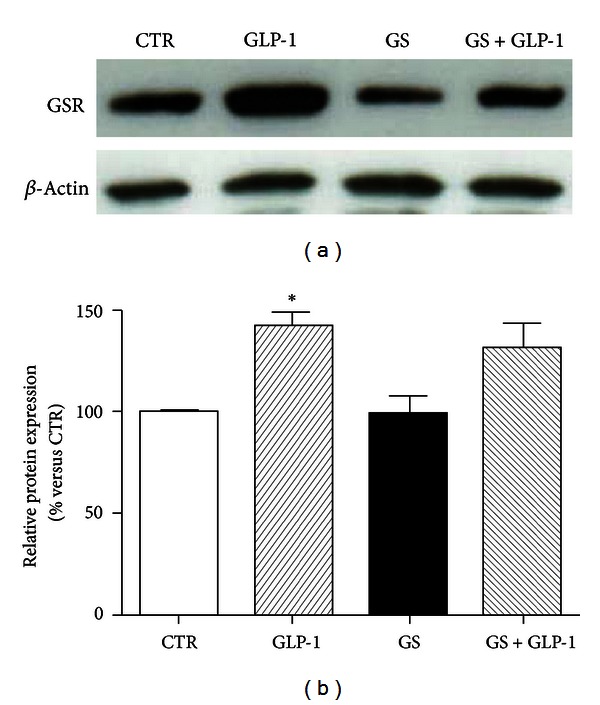
GLP-1 does not affect GSR expression in HIT-T15 cells cultured with AGEs. (a) Representative western blot of three different experiments. (b) Quantification of densitometries of western blot bands. Data were expressed as mean ± SE of fold induction relative to *β*-actin (*n* = 3). *P < 0.05 versus CTR.

We have recently shown that incubation with GS alters oxidative stress and the availability of the reduced form of glutathione (GSH) in the same culture model of pancreatic beta-cells [11]. Since lower levels of GSH were found in mice lacking the transcriptional repressor Nrf2 [24], which is implicated in the regulation of detoxification enzymes [25], we investigated whether Nrf2 expression (both mRNA and protein) was affected by GS and/or GLP-1. mRNA expression of Nrf2 was not affected by the incubation with GS in the presence or absence of GLP-1 (Figures 2(a) and 2(b)). However, GLP-1 significantly upregulated the protein expression of Nrf2 (Figures 2(c) and 2(d)) in cells cultured with GS for 5 days. Since Nrf2 has been shown to maintain GSH homeostasis by upregulating the expression of GSR [19], we further investigated whether GS and GLP-1 might affect expression of GSR. Our results showed that GS did not affect GSR protein expression (Figures 3(a) and 3(b)). On the other hand, GLP-1 increased GSR protein expression in both control and GS-treated cells. However, the statistical analysis revealed a significant increase only for control cells (Figures 3(a) and 3(b)).

### 3.3. GLP-1 Restores MafA and PDX-1 Levels in Pancreatic Beta-Cells Exposed to GS

**Figure 4:**
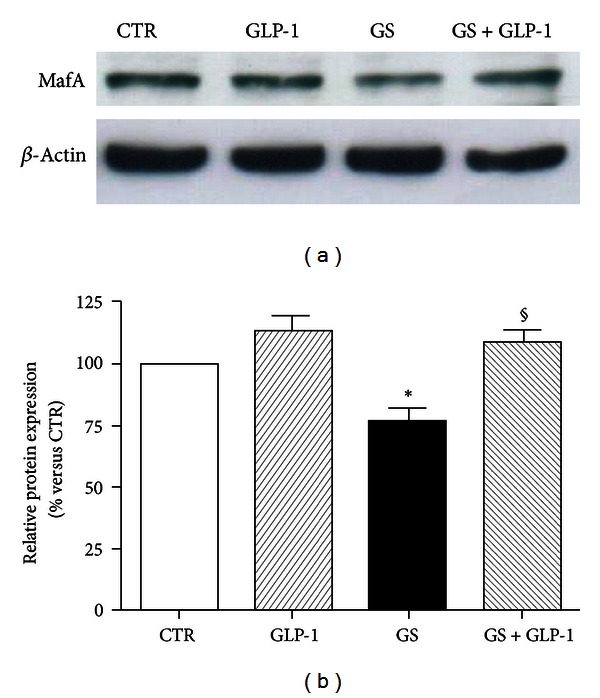
GLP-1 increases MafA protein expression levels in HIT-T15 cells cultured in the presence of GS. (a) Representative western blot of three different experiments. (b) Quantification of densitometries of western blot bands. Data were expressed as mean ± SE of fold induction relative to *β*-actin (*n* = 3). *P < 0.05 versus CTR; ^§^ *P* < 0.05 versus GS.

**Figure 5:**
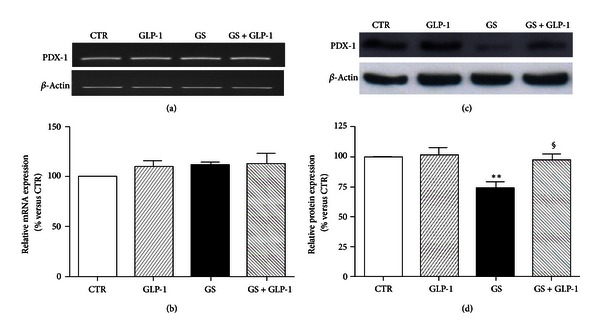
GLP-1 restores PDX-1 protein levels in HIT-T15 cells cultured in the presence or absence of AGEs. (a)-(b) Semiquantitative RT-PCR for PDX-1 mRNA expression in HIT-T15 cells cultured 5 days in standard medium (CTR) or in medium containing AGEs (GS) in the presence or absence of 10 nmol/L GLP-1. (a) Representative agarose gel of three different experiments. (b) Quantification of densitometries of agarose gel bands. Data were represented as percentage change from CTR level (100%) (*n* = 3). (c)-(d) Western blot analysis for PDX-1 protein intracellular expression on HIT-T15 cells, cultured in the same conditions of mRNA experiments. (c) Representative western blot of three different experiments. (d) Quantification of densitometries of western blot bands. Data were expressed as mean ± SE of fold induction relative to *β*-actin (*n* = 3). **P < 0.01 versus CTR; ^§^ *P* < 0.05 versus GS.

**Figure 6:**
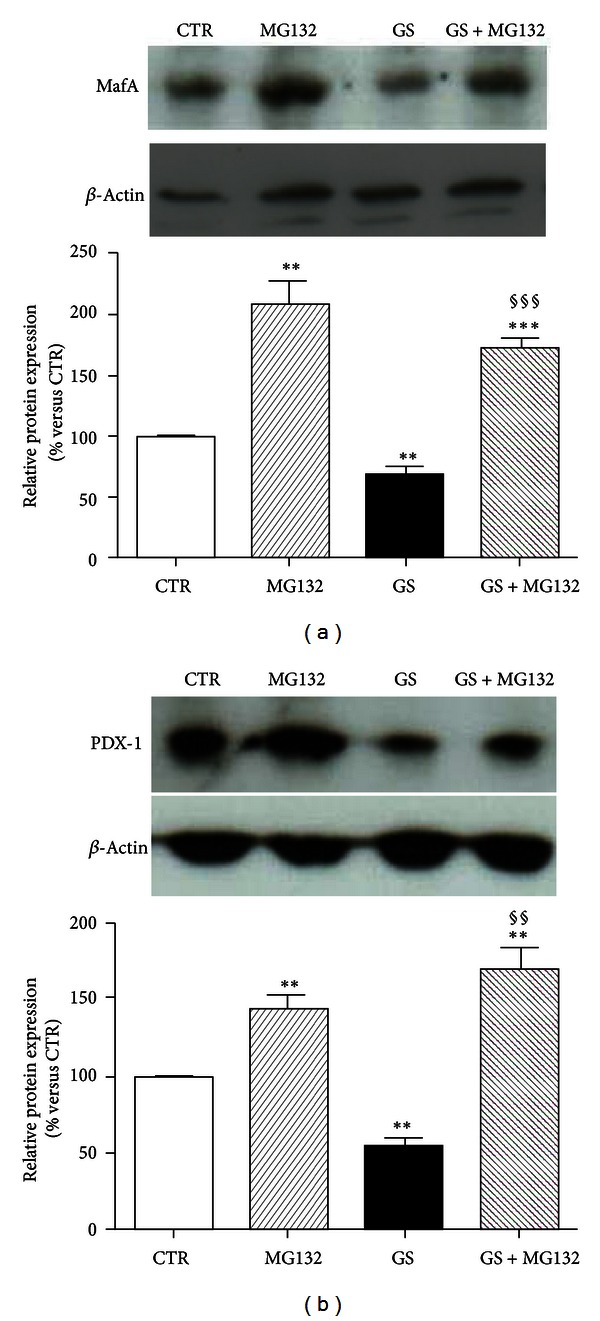
Pharmacological inhibition of proteasome increased accumulation of MafA and PDX-1. HIT-T15 cells were cultured for the last 15 h in the presence or absence of the proteasome inhibitor MG-132, and whole cell extracts were analyzed by western blotting using specific antibodies against PDX-1 and MafA. Actin amounts were analyzed as a loading control. *P < 0.05, **P < 0.01 and ***P < 0.001 versus CTR or versus the same condition without MG132.

**Figure 7:**
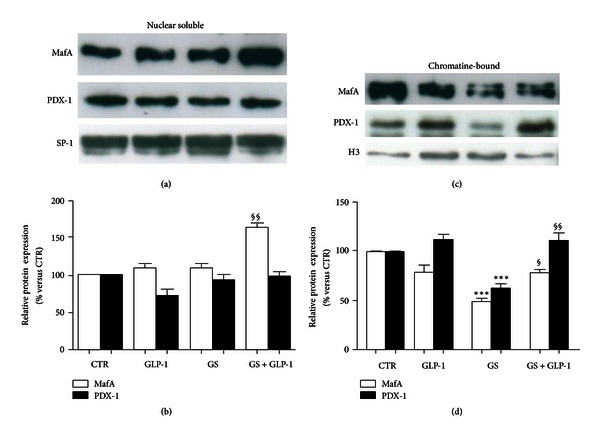
Effects of AGEs on nuclear distribution of MafA and PDX-1. Western blot analysis of nuclear soluble (a-b) and chromatin-bound (c-d) localization of MafA and PDX-1. (a) Representative western blot of three different experiments. SP-1 amounts were analyzed as a loading control. (b) Quantification of densitometries of western blot bands. Data were represented as percentage change from CTR level (100%) (*n* = 3). (c) Representative western blot of three different experiments. Histone H3 (H3) amounts were analyzed as a loading control. (d) Quantification of densitometries of western blot bands. Data were represented as percentage change from NGS level (100%) (*n* = 3). ***P < 0.001 versus CTR; ^§^ *P* < 0.05 and ^§§^ *P* < 0.01 versus GS.

Oxidative stress-induced damage has been shown as related to the downregulation of the endocrine transcription factors MafA and PDX-1 [26].  Figure 4 showed that a glycated environment (GS) significantly decreased MafA expression. Coincubation with GLP-1 to the GS restored MafA protein expression (Figures 4(a) and 4(b)). GLP-1 also improved the downregulation of PDX-1 induced by GS (Figure 5). Although no statistically significant effect was observed at mRNA expression level (Figures 5(a) and 5(b)), GLP-1 completely reverted the GS-mediated reduction of PDX-1 at protein level (Figures 5(c) and 5(d)). The discrepancy between gene and protein expression observed in the results shown above suggests the involvement of a posttranslational mechanism. The ubiquitin-proteasome system is the main mechanism degrading intracellular altered proteins and it might be altered by AGEs [27]. To verify this hypothesis, we evaluated the levels of PDX-1 and MafA in HIT-T15 cells treated for the last 15 h in the presence or absence of the proteasome inhibitor MG-132. The low concentration of MG-132 (150 nmol/L) and reduced time of exposure (15 h) were selected to avoid cytotoxicity [28]. As shown in Figure 6, treatment of cells with MG-132 induced accumulation of PDX-1 and MafA within the cells. Furthermore, the nuclear distribution of MafA and PDX-1 was also investigated (Figure 7). We found that GS did not affect distribution of MafA in the nuclear soluble compartment but reduced the immunoreactivity of MafA in the chromatin-bound fractions (Figures 7(c) and 7(d)). GLP-1 increased both nuclear soluble and chromatin-bound fraction of MafA in cells cultured with GS (Figure 7). Stimulation with GS reduced the presence of PDX-1 in the chromatin-bound fractions as compared to control CTR (Figures 7(c) and 7(d)). Coincubation with GLP-1 selectively increased immunoreactivity of PDX-1 in the chromatin-bound fractions of GS-cultured cells (Figures 7(c) and 7(d)).

### 3.4. GLP-1 Reverses GS-Induced Reduction of Proinsulin Production in Pancreatic Beta-Cells

**Figure 8:**
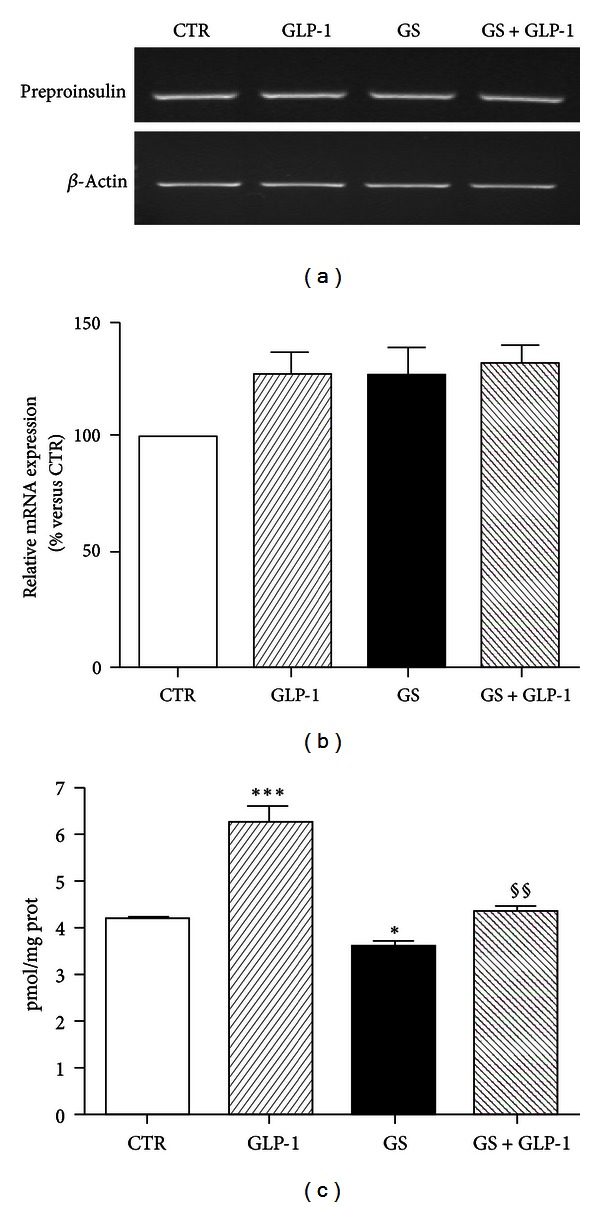
GLP-1 restores proinsulin content in HIT-T15 cells. (a) Semiquantitative RT-PCR for preproinsulin. Representative agarose gel of three different experiments. (b) Quantification of densitometries of agarose gel bands. Data are represented as percentage change from CTR level (100%) (*n* = 3). (c) Intracellular proinsulin was measured after acidified ethanol extraction. Data were expressed as mean ± SE of at least 3 independent experiments. **P < 0.01 and ***P < 0.001 versus CTR; ^§§^ *P* < 0.01 versus GS.

We recently demonstrated that exposure to GS decreased insulin content in HIT-T15 cells [12, 29]. To assess whether the GLP-1-mediated improvements on expression and subcellular localization of MafA and PDX-1 were associated with the increase in proinsulin production, we evaluated preproinsulin gene expression and proinsulin content. GS did not affect preproinsulin mRNA expression (Figures 8(a) and 8(b)). However, incubation with GS decreased proinsulin content (Figure 8(c)). GLP-1 significantly increased proinsulin content in HIT-T15 cells treated in standard condition (CTR) or in the presence of GS (Figure 8(c)).

### 3.5. Loss of GLP-1R Expression Abrogates GLP-1-Mediated Effects

**Figure 9:**
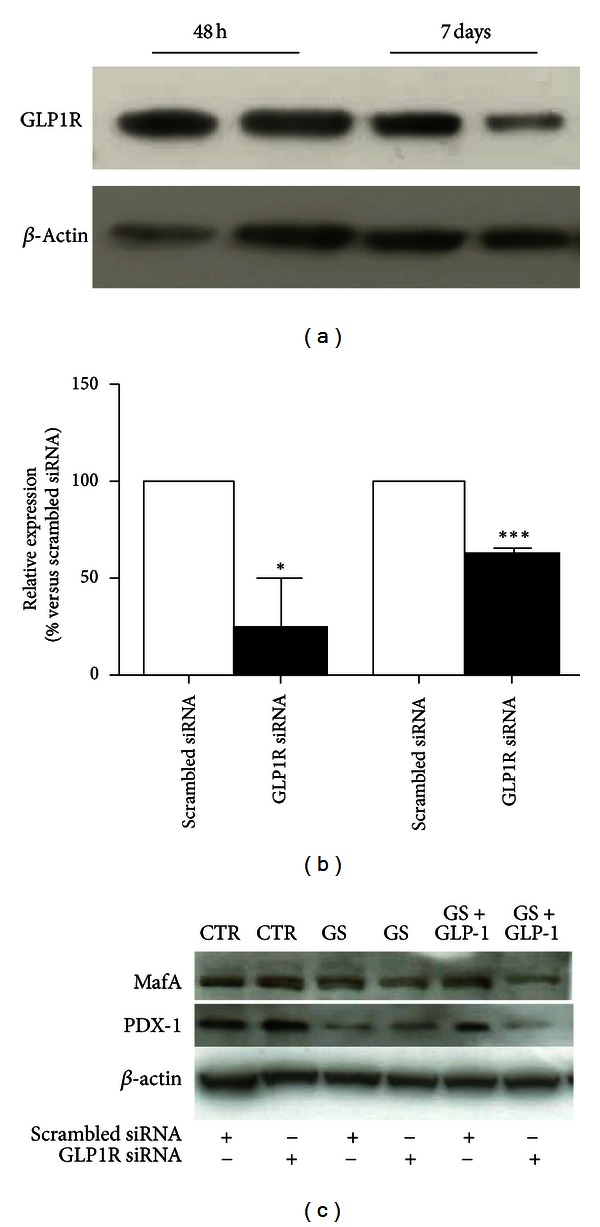
GLP-1R downregulation in HIT-T15 cells. HIT-T15 cells were transfected with specific siRNA for GLP-1R (GLP-1R-siRNA) or a random sequence (scrambled-siRNA). (a) Expression of GLP-1R was analyzed 48 hours and 7 days after transfection. (b) Transfection of HIT-T15 cells with GLP-1R siRNA results in more than 50% knockdown of GLP-1R expression. (c) Five days after treatment with GS, cells were lysed and immunoblotted with specific antibodies against MafA, PDX-1, and *β*-actin proteins. Blots are representative of three independent experiments. *P < 0.05 and ***P < 0.001 versus scrambled siRNA.

To confirm that GLP-1-mediated amelioration of GS-induced damage is mediated through the interaction with its transmembrane receptor, the expression of GLP-1R was downregulated by siRNA. Expression of GLP-1R was analyzed at the beginning (48 hours after transfection) and at the end of the treatment (7 days after transfection). As shown in Figures 9(a) and 9(b), transfection of HIT-T15 cells with GLP-1R siRNA results in more than 50% knockdown of protein content up to 7 days after transfection. Then, we investigated the beneficial effects induced by GLP-1 on the expression of PDX-1 and MafA. The results showed that downregulation of GLP-1R expression prevented the protective effects of GLP-1 on GS-induced depletion of PDX-1 and MafA (Figure 9(c)).

## 4. Discussion

We stimulated cells in the presence of GS (consisting in a pool of AGEs), showing that the detrimental effects of GS on the pancreatic beta-cell line HIT-T15 are due to the deprivation of proteins involved in the maintenance of the redox balance, and to the downregulation of transcription factors of the insulin gene. Furthermore, we showed that GLP-1 was able to counteract AGE-mediated dysfunction, restoring selective pathways regulating proinsulin production in pancreatic beta-cells.

We previously reported that AGE-induced pancreatic beta cell dysfunction was associated with an increase in oxidative stress [11, 12]. In the present paper, we found that GS increased ROS production and reduced Nrf2 expression. These findings suggest that AGE-induced damage in pancreatic beta-cells is not only due to ROS overproduction, but also to an increased susceptibility to oxidative stress. Furthermore, we showed that GLP-1 upregulated the protein expression of Nrf2 and GSR (the enzyme converting GSSG to GSH), thus resulting in an improvement of the antioxidant response. Since we recently showed that AGEs increased GSSG and decreased GSH levels in pancreatic beta-cells, it could be possible that GLP-1-mediated protection might prevent redox imbalance increasing GSH availability [11]. In addition, the molecular pathway responsible for GLP-1-mediated increase of GSH levels might be related to the upregulation of Nrf2 expression. The production of ROS has been often associated with the decrease in the nuclear availability of MafA and PDX-1, two transcription factors, which synergistically activate the insulin gene promoter [30]. Importantly, it has been reported that AGEs reduce insulin synthesis in pancreatic beta-cells by decreasing expression of PDX-1 through repression of PDX-1 protein expression at the posttranslational level [29, 31]. According to these findings, we showed that GS did not affect mRNA expression level of PDX-1 but decreased the intracellular protein. Similar results were showed also for MafA protein levels. We found that the pharmacological inhibition of proteasome resulted in the prevention of PDX-1 and MafA depletion in cells cultured in the presence of GS, confirming that AGEs may reduce PDX-1 and MafA protein expression targeting proteasome degradation. Furthermore, our results indicate that GS also decreased the ability of these transcriptional factors to bind DNA. Indeed, we found that GS decreased the immunoreactivity of these transcription factors in the chromatin-bound fraction, suggesting that, although they still localized in the nucleus, their activity might be reduced. These results are in accordance with those of Poitout and coworkers, reporting that glucotoxicity and lipotoxicity inhibited insulin expression through reduction of both DNA-binding activity and protein levels of MafA and PDX-1 [32]. Since it has been reported that insulin gene expression can be regulated by epigenetic mechanisms and that methylation of the insulin promoter negatively correlates with insulin gene expression [33], our results suggest that AGEs may alter DNA making it less accessible to transcription factors of the insulin gene. Despite these detrimental GS-mediated effects on MafA and PDX-1, we did not observe any reduction in preproinsulin mRNA expression levels. These results suggest that the residual binding activity of MafA and PDX-1 might be sufficient for maintaining acceptable levels of insulin gene transcription. Similarly to PDX-1, the discrepancy between conserved mRNA expression and decreased proinsulin content suggests the involvement of a posttranslational mechanism. Given a high secretory demand, the endoplasmic reticulum (ER) is very well developed and highly active in pancreatic *β*-cell. This also likely increases the susceptibility of these cells to ER stressors, which might produce signals mediating glucose-induced impairment of cell function and death. Experimental evidence indicated a role of ER stress in the progressive reduction of insulin secretion [34–36]. Since GLP-1R ligands have been described to potentiate the expression of proteins involved in the response to ER stress [37], GLP-1/GLP-1R might directly trigger ER protective pathways restoring proinsulin content. Importantly, we showed that coincubation with GLP-1 selectively restored these insulin-related transcriptional pathways. Indeed, GLP-1 treatment was also associated with an improvement in the binding to the chromatin of these transcription factors. Given the silencing experiments, GLP-1-mediated beneficial effects were due to its binding to GLP-1R on cell surface. These results demonstrated that GLP-1 via its transmembrane receptor might ameliorate the DNA accessibility to the protective transcriptional factors, thus reversing GS-induced pancreatic beta-cell injury.

Although HIT-T15 cells are among the most widely used insulin-secreting cell lines and might provide valuable information about both physiological and pathophysiological processes [38], their use represents a limitation of this study. In fact, the use of a cell line represents a simplified in vitro model that is characterized by low variation between experiments and potentially different from in vivo pancreatic islets or primary beta-cell pathophysiology. Another limitation might be represented by the use of GS that might contain artificial AGEs due to the preparation method. These points render our conclusions as highly speculative and potentially might not reflect the in vivo human pathophysiology.

## 5. Conclusions

We provided evidence that GLP-1 protects against the detrimental effects of GS on insulin production in the HIT-T15 pancreatic beta-cell line. GLP-1 has been shown to potently counteract ROS release and prevent transcriptional factor level reduction as well as pancreatic beta cell dysfunction. GLP-1 might be considered as a potential antagonist of the acceleration of pancreatic beta-cell deterioration in the presence of GS.

## Figures and Tables

**Table 1:** DNA sequences of the sense and antisense primers for RT-PCR analysis and cycler conditions.

Gene	Accession number (GeneBank)	Primer sequences	Product size (bp)	Annealing temperature (°C)	Number of cycles
Preproinsulin	XM_003508080.1	5′-*CTTTTGTCAAACAGCACCTTTGTG-*3′* (sense)* 5′-*TCAGTTGCAGTAGTTCTCCAGTTGG-*3′* (antisense) *	263	48	25
PDX-1	XM_003496658.1	5′-*CAGCTCCCTTTCCCGTGG A-*3′* (sense)* 5′-*GAGCATCACTGCCAGCTCCA-*3′* (antisense) *	204	55	28
Nrf2	XM_003498316.1	5′-*CTCACTGGATAAAGAAGTC-*3′* (sense)* 5′-*CCGCCCAGAAGTTCAGAGA-*3′* (antisense) *	240	60	40
*β*-Actin	NM_031144.2	5′-*AAGAGAAGCTGTGCTATGTTGC-*3′* (sense)* 5′-*CCTTGATCTTCATGGTGCTAGG-*3′* (antisense) *	324	48	25

PDX-1, Pancreatic and duodenal homeobox 1; Nrf2, Nuclear factor (erythroid-derived 2)-like 2.

## Glossary

AGEs: Advanced glycation end products
GLP-1: Glucagon-like peptide-1
GLP-1R: Glucagon-like peptide-1 receptor
PDX-1: Pancreatic and duodenal homeobox-1
MafA: v-Maf musculoaponeurotic fibrosarcoma oncogene homologue A
GSH: Glutathione
GSSG: Glutathione disulfide
Nrf2: Nuclear factor erythroid-derived 2
GSR: Glutathione reductase
FBS: Foetal bovine serum
GS: Glycated serum
NGS: Nonglycated serum
PBS: Phosphate saline buffer
RT-PCR: Reverse transcriptase polymerase reaction
siRNA: Short-interfering RNA.
